# Depressed mood during the menopausal transition: is it reproductive aging or is it life?

**DOI:** 10.1186/s40695-017-0030-x

**Published:** 2017-12-11

**Authors:** Ellen Sullivan Mitchell, Nancy Fugate Woods

**Affiliations:** 1 Family and Child Nursing, University of Washington, Seattle, USA; 2 Biobehavioral Nursing and Health Informatics, University of Washington, Seattle, USA

**Keywords:** Depressed mood, Menopausal transition stages, Early postmenopause, Urinary estrone, Health-related factors, Stress factors, Social factors, Abuse history, Sleep disruption symptoms

## Abstract

**Background:**

Although there has been noteworthy attention to both depressed mood symptoms and majordepressive disorder during the menopausal transition (MT), recently investigators have questioned whether there is an over-pathologizing of the MT by emphasizing hormonal effects on depression and deflecting attention from the everyday conditions of women’s lives as they relate to depressed mood. In addition, fluctuation of mood over short periods of time may not be captured by measures of depressed mood symptoms such as the CESD, especially when administered using a reference period such as a week or more. The purpose of this study was to examine the association of menopausal transition factors, health-related factors, stress factors, social factors and symptoms with repeated measures of depressed mood reported for a 24 h period.

**Methods:**

Seattle Midlife Women’s Health Study participants (*n* = 291, 6977 observations) provided data from 1990 to 2013 including annual questionnaires, symptom diaries and urine specimens assayed for hormones several times per year. Multilevel modeling was used to test bivariate and multivariable models accounting for depressed mood severity.

**Results:**

In individual models with age as the measure of time, being in early postmenopause, exercising more, and being partnered were associated with less severe depressed mood; greater perceived stress, having a history of sexual abuse, difficulty getting to sleep, early awakening, and awakening at night were each associated with higher depressed mood severity. In a multivariable model (*n* = 234, 6766 observations), being older, being in the early postmenopause, exercising more, being partnered, were associated with less severe depressed mood; reporting greater perceived stress, history of sexual abuse, difficulty getting to sleep and early awakening were associated with more severe depressed mood.

**Conclusions:**

Clinicians need to consider the context in which midlife women experience the menopausal transition and mood symptoms as well as hormonal transitions during this part of the lifespan.

## Background

Gender differences in reports of depressed mood, as well as episodes of major depressive disorder, have prompted investigators to examine periods of biological variability in women’s lives, such as phases of the menstrual cycle, pregnancy and postpartum, and the menopausal transition (MT), as windows of vulnerability [1, 2]. Progress in understanding stages of reproductive aging has provided a framework for understanding biological variability during the menopausal transition and early postmenopause as related to depression and depressed mood [3–7].

Longitudinal studies of community-based cohorts experiencing transitions of reproductive aging, including the Melbourne Midlife Women’s Health Project (MMWHP) [8], the Study of Women’s Health Across the Nation SWAN) [9], the Penn Ovarian Aging Study (POAS) [10], and the Seattle Midlife Women’s Health Study (SMWHS) [11] included data on a variety of measures of depressed mood symptoms from multi-ethnic community-based cohorts of women studied annually (some more frequently) for 20 years or longer. These studies have revealed a pattern of increasing depressed mood symptoms during the menopausal transition. Massachusetts Women’s Health Study investigators had documented an increase in depressive symptoms, measured by the Center for Epidemiologic Studies Depression Scale (CES-D), especially among women who experienced a lengthy MT [12]. SWAN investigators found that dysphoric mood symptoms, measured by 4 mood symptoms, increased during early MT [13]. The Harvard Study of Moods and Cycles investigators found an elevated odds ratio (2.5) of experiencing depressive symptoms (CES-D) during the MT vs premenopause (late reproductive stage) [14] and POAS investigators reported elevated odds ratios of 1.5 to 5.4, depending on women’s past history of depression [15, 16]. In addition, evidence supports a decrease in negative mood as women transition to early postmenopause. MMWHP investigators found an improvement in negative mood, measured by 10 negative adjectives, during the late MT stage and a decrease in negative mood as women progressed to PM [17]. POAS investigators found that reaching the final menstrual period (FMP) played a pivotal role in reduced prevalence of depressive symptoms (CES-D) [18].

Patterns of increasing incidence of major depressive disorder mirror those of depressed mood symptoms. SWAN investigators studying major depressive disorder found an increase in prevalence of new depression during the MT and early PM [19, 20].

Given the relationship seen between reproductive aging stages and depressed mood, it is tempting to attribute this association to hormonal changes. Investigations of the relationship of estrogen, testosterone, DHEAS, inhibin B, LH and FSH levels and variability to depressed mood have yielded a lack of definitive conclusions, likely attributable to variability in the endocrine measures, their timing and frequency, variability in the depressed mood measure and various analytic strategies used to assess hormone effects [21]. Nonetheless, there is some evidence implicating some hormones: POAS investigators found evidence for effects of increased levels of estradiol, FSH, decreased levels of inhibin B and LH, and greater estradiol, LH, and FSH variability on depressive symptoms (CES-D) [16, 17]. SWAN investigators found effects of both testosterone levels and their increase during the MT on depressive symptoms, but no associations between FSH or estradiol levels and their changing levels with depressive symptoms or with major depressive disorder [22, 23]. MMWHP investigators found declining estradiol levels were associated with depressive symptoms in a sample of women studied primarily during the PM [24].

The SMWHS team analyzed factors influencing depressive symptoms measured annually with the CES-D, finding no significant relationships with urinary estrone, FSH, testosterone, or cortisol [25, 26]. Instead, depressive symptoms were a function of age (being younger) and being in late MT stage when the severity of depressive symptoms increased. Hot flashes, life stress, family history of depression, history of postpartum blues, sexual abuse history, BMI, and use of antidepressants were also related to depressive symptoms. Age at entry into and duration of late MT stage were unrelated to depressive symptoms. These findings suggest that factors accounting for depressive symptoms earlier in the life span, as well as contemporary stressors, influenced depressive mood symptoms during the MT.

Although there has been noteworthy attention to both depressed mood symptoms and major depressive disorder during the MT, recently investigators have questioned whether there is an over-pathologizing of the MT by emphasizing hormonal effects on depression and deflecting attention from the everyday conditions of women’s lives as they relate to depressed mood [27]. In addition, fluctuation of mood over short periods of time may not be captured by measures of depressed mood symptoms such as the CESD, especially when administered using a reference period such as a week or more. Indeed, investigators have recently used ecological momentary assessment, a data capture technique involving repeated sampling made close in time to the experience in naturalistic environments [28]. Moore and colleagues found that among adults 65 years of age and older, sensitivity to change of the same symptoms of depressed mood, anxiety, and mindfulness varied across two different assessment methods: ecological momentary assessment (EMA) and traditional paper and pencil measures. Indeed, results indicated greater sensitivity of the ecological momentary assessment measures of depression, anxiety, and mindfulness in response to a mindfulness-based stress response intervention than a control intervention when the same symptoms were reported using the same items administered by EMA and by paper and pencil measures with a one week reference period.

**Fig. 1:**
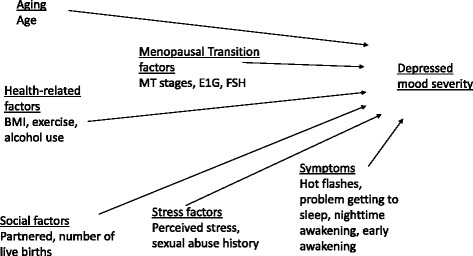
Factors influencing depressed mood severity during the menopausal transition and early postmenopause

The SMWHS team had obtained a single symptom measure of depressed mood, defined as an emotional state experienced over the past 24 h (reference period was today), on multiple occasions throughout multiple years of the study. Overnight urine samples were provided on the same day as the depressed mood rating and assayed for a variety of endocrine measures (e.g. urinary estrone-3-glucuronide (E1G)) [25]. The purpose of the analyses reported here was to test a longitudinal model of the effects of MT factors (menopausal transition stages, estrone, FSH), health-related factors (alcohol use, BMI, amount of exercise), stress factors (perceived stress, history of sexual abuse), social factors (partner status, number of live births), and symptoms (hot flashes and sleep symptoms) on depressed mood severity reported for a 24 h period. (See Fig. 1). We hypothesized that depressed mood would be positively related to perceived stress, history of sexual abuse, hot flashes, sleep symptoms, BMI, alcohol use, and FSH levels, and negatively related to age, being in the early postmenopausal stage, estrone levels, amount of exercise, being partnered, and number of live births.

## Methods

### Design

The data for these analyses are from a longitudinal study of the MT, the Seattle Midlife Women’s Health Study (SMWHS), described in greater detail elsewhere [29]. Women entered the cohort between 1990 and early 1992 when most were not yet in the MT or were in the early stages of the transition to menopause. Eligibility for the parent study included ages 35–55, at least one ovary, a period within the previous 12 months, not pregnant or lactating and able to speak and read English. Screening all households within selected multi-ethnic neighborhoods in Seattle (11,222 households) yielded 820 women eligible for the study and 508 were able to participate in an interview during the recruitment window. After completing an initial in-person interview (*n* = 508) administered by a trained Registered Nurse interviewer, participants (*n* = 390) began providing data annually by questionnaire, menstrual calendar, and health diary. The health diary included a symptom checklist that included depressed mood and other symptoms, as well as indicators of health behaviors and stress. Diary data were obtained on days 5, 6 and 7 of the menstrual cycle and a first morning voided urine specimens was collected on day 6. The women provided urine specimens 8 to 12 times per year for endocrine assays (from late 1996 through 2000), and then quarterly for 2001–2005. These data were in addition to an annual health questionnaire and menstrual calendars.

### Sample

Eligible participants for this study (*N* = 291) were those who contributed ratings of depressed mood severity from the health diaries beginning in 1990 and were in either the late reproductive, early or late MT stages, or within 5 years from FMP at data collection during the course of the study. Women not eligible for this study either did not keep a daily diary or were not able to be classified into one of the eligible stages due to hormone use, inadequate calendar data, hysterectomy, chemotherapy or radiation therapy.

Women eligible for inclusion had a mean age of 41.5 (SD = 4.3) years at the beginning of the study, 15.9 years of education (SD = 2.8), and a median family income of $38,200 (SD = $15,000). Most (87%) were currently employed, 71% married or partnered, 22% divorced or separated or widowed, and 7% never married or partnered. They described their racial/ethnic identity as African American (7%), Asian American (9%), and White (82%). Women included in these analyses were similar with respect to employment status, marital status, and age to those who were ineligible. The only significant differences between those who were included and those who were not were higher incomes, greater likelihood to be White, and more years of education than those in the study. (See Table 1). Data obtained on any occasion when the woman was using hormones were excluded.

**Table 1:** Sample characteristics at start of study (1990–1991) of the eligible and ineligible women in the mixed effects modeling analyses of depressed mood severity

Characteristic	Eligible Women	Ineligible Women	*p* value*
	(*n* = 291)	(*n* = 217)	
	Mean (SD)	Mean (SD)	
Age (years)	41.5 (4.3)	42.0 (5.0)	0.18
Years of education	15.9 (2.8)	15.3 (3.0)	0.03
Family income ($)	38,200 (15,000)	35,200 (17,600)	0.04
BMI wt kg/ht. m^2^)	25.3 (5.4)	27.1 (7.2)	0.002
Exercise (min/week)	187.8 (286.9)	179.0 (368.5)	0.76
Characteristic	*N* (Percent)	*N* (Percent)	*p* value**
Currently employed			0.42
Yes	254 (87.3)	184 (84.8)	
No	37 (12.7)	33 (15.2)	
Race/ethnicity			0.001
African American	20 (6.9)	38 (17.5)	
Asian /Pacific Islander	27 (9.3)	16 (7.4)	
White	238 (81.8)	153 (70.5)	
Other (Hispanic, Mixed)	6 (2.1)	10 (4.6)	
Marital Status			0.20
Married/partnered	278 (71.1)	141 (65.0)	
Divorced/widowed/not partnered	63 (21.7)	62 (28.6)	
Never married/partnered	21 (7.2)	14 (6.5)	
Alcohol Use			0.001
Yes	264	174	
No	26	42	

*Independent t-test

**Chi-square test

### Measures

Measures used in these analyses include: age; MT-related factors (MT stages, urinary estrone and urinary FSH); health-related factors (BMI, alcohol use and amount of exercise); stress factors (perceived stress and history of sexual abuse), social factors (number of live births and partner status), symptoms (hot flash severity and sleep symptoms) and depressed mood severity.

#### Depressed mood severity

Depressed mood severity was assessed in the health diary using one item that asked women to rate the severity of their feeling “depressed/sad or blue” on a scale where 1 indicated not present, 2 mild, 3 moderate and 4 extreme. The reference period was for “today”. This single item has been validated in the PROMIS measures [30–32].

#### Menopausal transition-related factors

Menopausal transition-related factors included MT stages as well as urinary estrone glucuronide and FSH. Using menstrual calendar data, women not taking any type of estrogen or progestin were classified according to stages of reproductive aging: late reproductive, early MT, late MT, or early PM, based on staging criteria developed by Mitchell, Woods and Mariella [11] and validated by the ReSTAGE collaboration [3–5]. The names of stages match those recommended at the Stages of Reproductive Aging Workshop (STRAW) [6]. The time before the onset of persistent menstrual irregularity during midlife was labeled the late reproductive stage when cycles were regular. Early stage was defined as persistent irregularity of 7 or more days absolute difference between any two consecutive menstrual cycles during the calendar year, with no skipped periods. Late stage was defined as persistent skipping of one or more menstrual periods. A skipped period was defined as 60 or more consecutive days of amenorrhea during the calendar year [3–5]. Persistence means the event, an irregular cycle or skipped period, reoccurred one or more times in the subsequent 12 months. Onset of early MT stage was the date of Day 1 for the bleeding segment when the irregularity criterion was first met. Onset of late stage was the date of Day 1 of the first bleeding segment with skipping. Final menstrual period was identified retrospectively after one year of amenorrhea without any known explanation. The first day of the FMP is synonymous with the term menopause and was used to determine age of onset of FMP. Early PM refers to the years within five years of the FMP.

#### Urinary assays

Urinary assays were performed in our laboratories using a first-voided morning urine specimen provided on day 6 of the menstrual cycle, if menstrual periods were identifiable. For women with no bleeding or spotting or extremely erratic flow, a consistent date each month was used. Women abstained from smoking, caffeine use, and exercise before the urine collection. All assays were adjusted for urinary creatinine which was assayed in urine specimens using the method of Jaffe [33]. More assay details are reported elsewhere [34].

#### Urinary estrone glucuronide (E_1_G)

Urinary estrone glucuronide (E_1_G) was selected to assess estrogens because it is stable, can be reliably measured without special preparation, and is highly correlated with serum estradiol levels [35–40]. Urinary E_1_G was measured by a competitive enzyme immunoassay (EIA) that cross-reacts 83% with estradiol glucuronide and less than 10% with free estrone, estrone sulfate, estriol glucuronide, estradiol and estriol [35]. The assay is described in detail elsewhere [41].

#### Urinary FSH

Urinary FSH was assayed using Diagnostic Products Corporation (DPC) Double Antibody FSH Kit, using a radioimmunoassay (RIA) was designed for the quantitative measurement of FSH in serum and urine [42]. The procedure is described in detail elsewhere [41].

#### Health-related factors

Health-related factors included women’s use of alcohol, amount of exercise and BMI. Women were asked to indicate in the daily health diary whether or not they drank alcohol on that day (coded as 0 for no and 1 for yes)**.** They estimated their exercise in response to the question: how many total minutes of non-work related exercise did you do today? (walking, running, biking, swimming, aerobics, sports, work out, gardening, yard work). In addition, height and weight were reported annually from which the BMI was calculated (wtkg^/^htm^2^).

#### Stress factors

Stress factors included perceived stress and history of sexual abuse. Perceived stress was assessed in the diary with a question “how stressful was your day?” that women rated from 1 (not at all) to 6 (extremely, a lot). Brantley, Waggoner, Jones, & Rappaport found that a global stress rating and the sum of stress ratings across multiple dimensions correlated significantly (*r* = .35, *p* < .01) [43] Sexual abuse history was assessed by asking “Have you ever been sexually assaulted, abused, or molested?” These data were obtained in 1999–2002. Also, beginning in 1996 and through the end of the study, women were asked “during the past year did you experience any sexual abuse or sexual assault?” A cumulative variable was created to represent any history of sexual abuse or assault and coded as 1 for yes and 0 for no at each data point.

#### Social factors

Number of live births and partner status were assessed yearly from two items in the annual health questionnaire as were income and employment status. Race/ethnicity was established by self-report at beginning of the study.

#### Symptoms

Hot flashes severity and sleep symptoms were assessed in the diary with single items that women rated from 0, not present to 4, extreme. Sleep symptoms included difficulty getting to sleep, awakening during the night, and early morning awakening.

### Analyses

To determine which covariates in the conceptual model (Fig. 1) had a significant effect when examined individually, bivariable mixed effects modeling was used. Finally, to test the multivariable model to determine which predictors had a significant effect on depressed mood severity over time, those individual variables from the bivariate analyses with a significance of *p* ≤ 0.1 were entered together in a multivariable mixed effects model [42–47].

Before testing the conceptual model, an initial series of statistical models tested age alone as a predictor of depressed mood severity to learn whether a random intercept, fixed slope model or a random intercept, random slope model was the best fit. It was first postulated that overall levels of depressed mood severity would differ from woman to woman (random intercept), but the scores would change with age in a common manner (fixed slope). The second statistical model extended the first to postulate that each woman had a different mean level of depressed mood severity and rate of change (random intercept, random slope). The best fitting model (fixed slope or random slope) was determined by using the maximum likelihood estimation with Akaike Information Criterion (AIC) [48].

Using the best fitting model, individual covariates were added iteratively to test the effect on depressed mood severity over time. Because these analyses were for exploration and to stimulate further mechanistic studies, a *p* value of ≤0.10 was used as the criterion of significance for the univariate model and ≤0.05 for the final model. Different numbers of women and observations occurred with each covariate tested because the analysis required pairing observations of the outcome and predictor variables at each time point. In all analyses, age was centered at the group mean to enable the interpretation of the effect of age on depressed mood severity.

## Results

Participants whose data were included in these analyses reported moderate levels of symptoms, with awakening during the night and problems getting to sleep, depressed mood, early awakening, and hot flashes in decreasing order of average severity. There were large standard deviations. Also 34% of these participants reported a history of sexual abuse. (See Table 2).

**Table 2:** Sample characteristics at start of study (1990–1991) of the eligible women in the mixed effects modeling analyses of depressed mood severity

Characteristic	Eligible Women
	Mean (SD)
Depressed mood severity (0–4)	0.41 (0.63)
Hot flashes (0–4)	0.11 (0.41)
Problem getting to sleep (0–4)	0.32 (0.67)
Awakening during the night (0–4)	0.57 (0.87)
Early morning awakening (0–4)	0.34 (0.66)
Perceived stress (1–6)	2.77 (1.09)
Characteristic	*N* (Percent)
History of sexual abuse (*N* = 233)	
Yes	80 (34%)
No	153 (66%)

With age as a measure of time, a random intercept, random slope model was used to test separately each covariate in the model for effect on depressed mood. Being in the early PM was significantly and negatively associated with depressed mood as was reporting a higher amount of exercise. In addition, perception of stress, history of sexual abuse, and each of the three sleep symptoms were associated with an increase in depressed mood. Being partnered was negatively associated with depressed mood. Age, estrone and FSH levels, BMI, alcohol intake, number of live births, and hot flash severity were not associated significantly with depressed mood. (See Table 3).

**Table 3:** Random effects models for depressed mood severity with age as predictor (β_2_) and with individual covariates (β_3_)

	Mean Values (*p* values)			Standard Deviations			Number	
Predictor	β_1_^a^	β_2_^a^	β_3_^a^	σ_1_^b^	σ_2_^b^	σ_ε_^b^	Women	Observations
Age (47.7)	0.51	<0.001 (0.84)	–	0.49	0.03	0.60	291	6977
Menopausal Transition Factors								
MT-stage	0.52 (<.001)	0.005 (0.25)		0.49	0.03	0.60	291	6977
Early			0.02 (0.56)					
Late			−0.006 (0.88)					
Early PM			−0.10 (0.05)					
Estrone (1.3)	0.62 (<.001)	−0.01 (0.04)	−0.03 (0.32)	0.50	0.04	0.60	131	4908
FSH (1.1)	0.58 (<.001)	−0.01 (0.04)	−0.02 (0.23)	0.50	0.04	.060	131	4996
Health-related factors								
BMI (26.0)	0.37 (<.001)	<0.001 (0.97)	0.005 (0.20)	0.49	0.03	0.60	291	6977
If drinks alcohol	0.51 (<.001)	<0.001 (0.84)	−0.001 (0.97)	0.49	0.03	0.60	291	6977
Amount of exercise	0.54 (<.001)	0.001 (0.69)	−0.001 (<.001)	0.49	0.03	0.60	291	6977
Social factors								
If partnered	0.58 (<.001)	<0.001 (0.87)	−0.11 (0.002)	0.49	0.03	0.60	291	6977
Number of live births	0.51 (<.001)	<0.001 (0.83)	−0.002 (0.93)	0.49	0.03	0.60	291	6977
Stress								
Perceived Stress	0.06 (0.10)	0.008 (0.02)	0.18 (<.001)	0.47	0.03	0.58	291	6977
History of sexual abuse	0.42 (<.001)	<0.001 (0.82)	0.25 (<.001)	0.47	0.03	0.60	234	6766
Symptoms								
Hot flashes	0.50 (<.001)	<0.001 (0.88)	0.003 (0.78)	0.49	0.03	0.60	291	6977
Problem getting to sleep	0.42 (<.001)	−0.0003 (0.91)	0.24 (<.001)	0.44	0.03	0.59	291	6977
Early awakening	0.44 (<.001)	−0.003 (0.41)	0.13 (<.001)	0.47	0.03	0.59	291	6977
Awaken at Night	0.43 (<.001)	−0.002 (0.50)	0.11 (<.001)	0.47	.03	0.59	291	6977

^a^β_1,_ β_2,_ β_3_ are the fixed effects (group averages) for the intercept, slope and covariate

^b^σ_1,_ σ_2,_ σ_ε_ are the random effects (variability) for the intercept, slope and residual error

When the significant factors (*p* ≤ 0.1) from the univariate analysis listed above were included in a multivariate model, perceived stress, history of sexual abuse, problem getting to sleep, and early awakening significantly predicted an increase in depressed mood while increased age, being in the early postmenopause, greater amount of exercise and being partnered were associated with a decrease in depressed mood. (See Table 4).

**Table 4:** Final random effects model for depressed mood (diary) with age as predictor and significant psychosocial and hormonal covariates entered simultaneously (*N* = 234; Observations = 6766)

	Beta Coefficient	Standard Error/Standard Deviation	*p* value
Fixed effects			
β_1_ intercept	0.05	0.05	0.34
β_2_ Age (−47.7) years	0.01	0.004	0.02
β_3_ Menopausal Transition Stage			
Early Stage	0.02	0.03	0.57
Late Stage	−0.01	0.04	0.77
Early PM	−0.10	0.05	0.03
Β_4_ Perceived Stress	0.16	0.008	<.001
Β_5_ History sexual abuse	0.18	0.06	0.002
Β_6_ Amount exercise	- < 0.001	0.0002	0.004
Β_7_ Problem getting to sleep	0.19	0.01	<.001
Β_8_ Early Awakening	0.05	0.01	<.001
Β_9_ Awakening at night	0.02	0.01	0.09
Β_10_ If partnered	−0.11	0.03	0.001
Random effects			
b_1_ Intercept σ_1_		0.39	
b_2_ Age (−47.7) years σ_2_		0.02	
b_ε_ residual σ_ε_		0.57	

## Discussion

In a multivariable model, depressed mood severity reported for a 24 h period was not associated with age, MT markers (reproductive aging stages, levels of estrone or FSH), or symptoms of hot flashes or awakening at night. Instead, factors reflecting the context of women’s lives, including perceived stress, history of sexual abuse, being partnered, amount of exercise, and sleep symptoms (early awakening, problem getting to sleep), were associated with severity of depressed mood.

In the analyses presented here, depressed mood severity reported for a 24 h period was not related to any of the MT markers. Although prior research has provided limited evidence linking endocrine changes and the MT stages to depressive symptoms and also to episodes of major depressive disorder among women with no prior history of depression, Freeman’s recent review concluded that the contribution of the changing endocrine milieu to the development of depressive symptoms was small [21]. She reminded us that only a minority of women experience debilitating depressive symptoms during this part of the lifespan and that hormonal change is not the only factor to consider in disentangling the complex pathways to depression.

There was not a significant relationship between hot flashes and depressed mood over the past 24 h in the diary-based data reported here. In contrast, Freeman’s review [21] indicated that although the association between hot flashes and depressive symptoms predominantly measured with the CES-D was confirmed, the direction of the relationship was unclear and evidence for a relationship between hot flashes and major depressive disorder was much less clear. Although hot flashes predicted negative mood on the next day in SWAN daily diary data, negative mood did not predict hot flashes on the subsequent day [49]. On the other hand, studies evaluating the risk of women with high levels of depressive symptoms (CES-D) indicated that depressive symptoms were likely to precede hot flashes among women who had not experienced either type of symptoms earlier in life [50, 51]. Women with consistently high levels of depressive symptoms in the SMWHS (CES-D) were more likely to experience hot flashes than those without more severe depressive symptoms [50]. Among POAS participants, 24% without high depressive symptoms (CES-D) or hot flashes at baseline reported depressive symptoms before reporting hot flashes over a 10 year follow up period, but only 8% reported hot flashes before reporting depressive symptoms [51].

Sleep symptoms and depressed mood co-occur frequently among midlife women. Indeed, use of poor sleep as one indicator of clinical depression makes it difficult to determine whether there is a causal relationship between the two symptoms. Awakening during the night was the only sleep symptom significantly related to MT stages in the SMWHS [52], but in the analyses reported here, both trouble getting to sleep and early awakening were associated with depressed mood over the past 24 h, while awakening during the night was not. Self-reported difficulty sleeping was associated with next-day negative mood in SWAN participants [49]. Burleson and colleagues tracked midlife women over 36 weeks during which they experienced the highest weekly frequency of vasomotor symptoms. They found that sleep problems occurring on one day predicted next-day negative mood (mean of 8 negative adjectives) more robustly than did vasomotor symptoms [53].

In a review of studies of sleep and the MT, Shaver [54] concluded that although the MT is associated with poor sleep beyond that anticipated with aging, perceptions of sleep are likely to be influenced by an emotional overlay on symptom reporting. Depressed mood and poor sleep are likely to be related in bidirectional ways [55]. Thus attention to mood as a factor contributing to sleep problems as well as sleep problems contributing to mood experiences is warranted. Induced poor sleep has been associated with more negative mood, but effects of improved sleep on mood during the MT remain to be examined. Induced poor sleep has been associated with more negative mood, but effects of improved sleep on mood during the MT remain to be examined. To date, a single clinical trial of cognitive behavioral therapy for insomnia delivered by telephone to women who were experiencing the menopausal transition or early postmenopause revealed that the treatment effect, when compared to a menopause education control, had the greatest treatment effects on hot flashes for women who had higher depression scores at baseline. Further analyses examining treatment effects on depressed mood are in process (McCurry, personal communication).

Perceived stress and stressful life events during MT have been associated with depressed mood in other cohorts [14, 23, 56]. SWAN investigators studied women’s experiences of stressful and very stressful life events. Using a scale of 18 items women rated as most stressful, they found that women in the MT experiencing two or more very stressful life events since the last study visit (usually a one year period) reported the highest depressive mood score [23]. More recently Gordon and colleagues found that reports of very stressful life events experienced by women in the MT during the six months before study baseline were associated with depressive symptoms (CES-D) over a year later [56]. Very stressful life events included only the following six events: divorce or separation from a partner, serious illness or death of a close family member or close friend, a major worsening in one’s financial status or major chronic financial problems, being physically attacked or having one’s life threatened, being sexually abused or assaulted, and being arrested for a serious crime or having a mate or close relative arrested for a serious crime [57]. Gordon and colleagues proposed that estradiol variability may have enhanced emotional sensitivity to these very stressful events that influenced depressed mood in this sample of midlife women 45–60 years of age [56, 58, 59].

The experience of sexual abuse has been included among very stressful life events in earlier studies and also associated with depressive symptoms in an earlier SMWHS investigation using the CES-D [25] as well as in the analyses of reports of depressed mood severity reported here. Sexual abuse history has not been associated with depressed mood in other cohorts of women during the MT. Nonetheless, Allsworth and colleagues found that history of sexual abuse or physical violence was associated with the timing of the MT [60] and violence history with high FSH and low estradiol during the perimenopause (MT), suggesting a plausible relationship of sexual abuse to endocrine perturbation [61].

Factors that protect women from experiences of depressed mood include those considered part of one’s health promotion practices, such as engaging in physical activity and being in positive relationships. Sternfield and colleagues found that midlife women participating in an exercise trial experienced less severe depressive symptoms than controls when measured by PHQ-8 [62]. Being in a relationship with a partner may serve as either a risk or protective factor depending on the nature of the relationship [25, 63]. In analyses reported here, being partnered had protective effects on depressive symptoms. It is likely that the nature of the relationship influences the experience of depressed mood: SWAN participants who reported stressful relationships with partners were likely to experience more depressive symptoms [63].

Limitations of this study include relatively small numbers of women who provided urine specimens for hormonal assays (*N* = 131) compensated for in part by the intensive measurement on multiple occasions, yielding over 4,900 data points. SWAN provided data from a larger number of women from multiple sites in the US with annual measures for most variables, including a daily hormone study involving nearly 1,000 women. In addition, the proportion of women in SMWHS with low levels of education and income and a low proportion of women of color limit our ability to generalize the results broadly. SWAN and the POAS included a much more representative population of women.

## Conclusions

In conclusion, given the frequent and coincident measures of depressed mood and endocrine levels available for our analyses, it is likely that estrone and FSH did not play a major role in depressed mood severity reported for the same 24 h period. Nonetheless, others have found relationships when analyses were focused on the MT stages or endocrine values and when using measures of MDD or more severe depressive symptoms such as those included in the CES-D and a longer reference period. In addition, the results reported here afforded an examination of the relationships among depressed mood, urinary estrone and FSH levels, and perceived stress, hot flashes and sleep symptoms obtained for the same 24 h time period as recorded in a health diary, conditions that have not been available to many other investigators. We conclude that it is important for clinicians to consider the relationship of depressed mood to everyday life, even during the MT when it is tempting to attribute depressed mood to endocrine variability. These findings bear replication in larger populations of women and those in which ethnic/racial variability is maximized.

## Abbreviations

FMP: final menstrual period
FSH: follicle-stimulating hormone
MT: menopausal transition
PM: Postmenopause
SMWHS: Seattle Midlife Women’s Health Study
STRAW: Staging Reproductive Aging Workshop

## References

[1] Pratt LA, Brody DJ (2014). Depression in the U. S. household population, 2009–2012. NCHS Data Brief.

[2] Deecher D, Andree TH, Sloan D, Schechter LE (2008). From menarche to menopause: exploring the underlying biology of depression in women experiencing hormonal changes. Psychoneuro.

[3] Harlow SD, Cain K, Crawford S, Dennerstein L, Little R, Mitchell ES, Nan B, Randolph JF, Taffe J, Yosef M (2006). Evaluation of four proposed bleeding criteria for the onset of late menopausal transition. J Clin Endocrinol Metab.

[4] Harlow SD, Mitchell ES, Crawford S, Nan B, Little R, Taffe J, ReSTAGE Collaboration (2008). The ReSTAGE collaboration: defining optimal bleeding criteria for onset of early menopausal transition. Fertil Steril.

[5] Harlow SD, Crawford S, Dennerstein L, Burger HG, Mitchell ES, Sowers MF, ReSTAGE Collaboration (2007). Recommendations from a multi-study evaluation of proposed criteria for staging reproductive aging. Climacteric.

[6] Soules MR, Sherman S, Parrott E, Rebar R, Santoro N, Utian W, Woods NF (2001). Executive summary: stages of reproductive aging workshop (STRAW). Fertil Steril.

[7] Harlow SD, Gass M, Hall JE, Lobo R, Maki P, Rebar RW, Sherman S, Sluss PM, de Villiers TJ (2012). Executive summary of STRAW+10: addressing the unfinished agenda of staging reproductive aging. Climacteric.

[8] Dennerstein L, Dudley EC, Hopper JL, Guthrie JR, Burger HG (2000). A prospective population-based study of menopausal symptoms. Obstet Gynecol.

[9] Sowers M, Crawford S, Sternfeld B, Morganstein D, Gold E, Greendale G, Evans D, Neer R, Matthews K, Sherman S, Lo A, Weiss G, Kelsye J, Lobo R, Kelsey J, Marcus R (2000). SWAN: a multicenter, multiethnic community-based cohort study of women and the menopausal transition. Menopause: biology and pathobiology.

[10] Freeman EW, Sammel MD, Lin H, Gracia ER, Pien GW, Nelson DB, Sheng L (2007). Symptoms associated with menopausal transition and reproductive hormones in midlife women. Obstet Gynecol.

[11] Mitchell ES, Woods NF, Mariella A (2000). Three stages of the menopausal transition from the Seattle midlife Women's health study: toward a more precise definition. Menopause.

[12] Avis NE, Brambilla D, McKinlay SM, Vass K (1994). A longitudinal analysis of the association between menopause and depression. Ann Epidemiol.

[13] Bromberger JT, Assmann SF, Avis NE (2003). Persistent mood symptoms in a multiethnic community cohort of pre-and perimenopausal women. Am J Epidemiol.

[14] Soares CN, Almeida OP (2001). Depression during the perimenopause. Arch Gen Psychiatry.

[15] Freeman EW, Sammel MD, Liu L, Gracia CR, Nelson DB, Hollander L (2004). Hormones and menopausal status as predictors of depression in women in transition to menopause. Arch Gen Psychiatry.

[16] Freeman EW, Sammel MD, Lin H, Nelson DB (2006). Associations of hormones and menopausal status with depressed in mood in women with no history of depression. Arch Gen Psychiatry.

[17] Dennerstein L, Guthrie JR, Clark M, Leher P, Henderson VW (2004). A population-based study of depressed mood in middle-aged, Australian-born women. Menopause.

[18] Freeman EW, Sammel MD, Boorman DW, Zhang R (2014). Longitudinal pattern of depressive symptoms around natural menopause. JAMA Psychiatr.

[19] Bromberger JT, Kravitz HM, Chang YF, Cyranowski JM, Brown C, Matthews KA (2011). Major depression during and after the menopausal transition: Study of Women’s Health Across the Nation (SWAN). Psychol Med.

[20] Bromberger JT, Kravitz HM, Matthews K, Youk A, Brown C, Feng W (2009). Predictors of first lifetime episodes of major depression in midlife women. Psychol Med.

[21] Freeman EW (2015). Depression in the menopause transition: risks in the changing hormone milieu as observed in the general population. Women’s Midlife Health.

[22] Bromberger JT, Schott LL, Kravitz HM, Sowers M, Avis NE, Gold EB (2010). Longitudinal change in reproductive hormones and depressive symptoms across the menpasual transition: results from the study of Women’s health across the nation (SWAN). Arch Gen Psychiatry.

[23] Bromberger JT, Matthews KA, Schott LL, Brockwell S, Avis NE, Kravitz HM (2007). Depressive symptoms during the menopausal transition: the study of Women’s health across the nation (SWAN). J Affect Disord.

[24] Ryan J, Burger HG, Szoeke C, Lehert P, Ancelin ML, Henderson VW (2009). A prospective study of the association between endogenous hormones and depressive symptoms in postmenopausal women. Menopause.

[25] Woods NF, Smith-DiJulio K, Percival DB, Tao EY, Mariella A, Mitchell ES (2008). Depressed mood during the menopausal transition and early postmenopause: observations from the Seattle midlife Women’s health study. Menopause.

[26] Woods NF, Smith-DiJulio K, Percival DB, Tao EY, Taylor HJ, Mitchell ES (2007). Symptoms during the menopausal transition and early postmenopause and their relation to endocrine levels over time: observations from the Seattle midlife Women’s health study. J Women's Health.

[27] Judd FK, Hickey M, Bryant C (2012). Depression and midlife: are we overpathologising the menopause?. J Affect Disord.

[28] Moore R, Depp CA, Wetherell JL, Lenze E (2016). Ecological momentary assessment versus standard assessment instruments for measuring mindfulness, depressed mood, and anxiety among older adults. J Psychiatr Res.

[29] Woods NF, Mitchell ES (2016). The Seattle midlife Women’s health study: a longitudinal prospective study of women during the menopausal transition and early postmenopause. Women’s Midlife Health.

[30] Bjorner J, Rose M, Gandek B, Stone A, Junghaenel D, Ware JJ (2013). Difference in method of administration did not significantly impact item response: an IRT-based analysis from the patient-reported outcomes measurement information system (PROMIS) initiative. Qual Life Res.

[31] Cella D, Yount S, Rothrock N, Gershon R, Cook K, Reeve B (2007). The Patient-Reported Outcomes Measurement Informatino System (PROMIS): progress of an NIH Roadmap cooperative group during its first two years. Med Care.

[32] Reeve B, Hays RD, Bjorner JB, Cook KF, Crane PK, Teresi JA (2007). Psychometric evalution and calibration of health-related quality of life item banks: plans for the Patient-Reported Outcomes Measurement Informatino System (PROMIS). Med Care.

[33] Taussky HH (1954). A microcolorimetric determination of creatinine in urine by the Jaffe reaction. J Biol Chem.

[34] Woods NF, Smith-DiJulio K, Percival DB, Tao EY, Taylor HJ, Mitchell ES (2007). Symptoms during the menopausal transition and early post menopause and their relation to endocrine levels over time: observations from the Seattle midlife Women’s health study. J Women's Health.

[35] Denari JH, Farinati Z, Casas PR, Oliva A (1981). Determination of ovarian function using first morning urine steroid assays. Obstet Gynecol.

[36] Stanczyk FZ, Miyakawa I, Goebelsmann U (1980). Direct radioimmunoassay of urinary estrogen and pregnanediol glucuronides during the menstrual cycle. Am J Obstet Gynecol.

[37] O'Connor KA, Brindle E, Holman DJ, Klein NA, Soules MR, Campbell KL, Kohen F, Munro CJ, Shofer JB, Lasley BL, Woods JW (2003). Urinary estrone conjugate and pregnanediol 3-glucuronide enzyme immunoassays for population research. Clin Chem.

[38] Baker TE, Jennison KIM, Kellie AE (1979). The direct radioimmunoassay of oestrogen glucuronides in human female urine. Biochem J.

[39] Ferrell RJ, O’Connor KA, Holman DJ (2005). Monitoring the transition to menopause in a five year prospective study: aggregate and individual changes in steroid hormones and menstrual cycle lengths with age. Menopause.

[40] O'Connor KA, Brindle E, Shofer JB, Miller RC, Klein NA, Soules MR, Campbell KL, Mar C, Handcock MS (2004). Statistical correction for non-parallelism in a urinary enzyme immunoassay. J Immunoass Immunochem.

[41] Woods NF, Mitchell ES (1997). Pathways to depressed mood for midlife women: observations from the Seattle midlife Women’s health study. Res Nurs Health.

[42] Qui Q, Overstreet JW, Todd H, Nakajima ST, Steward DR, Lasley BL (1997). Total urinary follicle stimulating hormone as a biomarker for detection of early pregnancy and peri implantation spontaneous abortion. Environ Health Perspect.

[43] Brantley PJ, Waggoner CD, Jones GN, Rappaport NB (1987). A daily stress inventory: development, reliability, and validity. J Behav Med.

[44] Pinheiro J, Bates D, DebRoy S, Sarkar D, R Core Team. nlme: Linear and nonlinear mixed effects models, R package version 3.1. 2107:1–131. https://CRAN.R-project.org/package=nlme.

[45] R Development Core Team (2005). R: A Language and Environment for statistical computing.

[46] Sarkar D. Lattice: multivariate data visualization with R. http://lattice.r-forge.r-project.org/.

[47] Pinheiro J, Bates D (2000). Mixed-effects models in S and S-PLUS.

[48] Hox J (2002). Multilevel analysis: techniques and applications.

[49] Gibson CJ, Thurston RC, Bromberger JT, Kamarck T, Matthews KA (2011). Negative affect and vasomotor symptoms in the study of Women’s health across the nation daily hormone study. Menopause.

[50] Woods NF, Mitchell ES (1996). Patterns of depressed mood in midlife women: observations from the Seattle midlife Women’s health study. Res Nurs Health.

[51] Freeman EW, Sammel MD, Lin H (2009). Tempral association of hot flashes and depression in the transition to menopause. Menopause.

[52] Woods N, Mitchell ES (2010). Sleep symptoms during the menopausal transition and early menopause: observations from the Seattle midlife Women's health study. Sleep.

[53] Burleson MH, Todd M, Trevathan WR (2010). Daily vasomotor symptoms, sleep problems, and mood: using daily data to evaluate the domino hypothesis in middle-aged women. Menopause.

[54] Shaver JL, Woods NF (2015). Sleep and menopause: a narrative review. Menopause.

[55] Kahn M, Sheppes G, Sadeh A (2013). Sleep and emotions: bidirectional links and underlying mechanisms. Int J Psychophysiol.

[56] Gordon JL, Rubinow DR, Eisenlohr-Moul TA, Leserman J, Girdler SS (2016). Estradiol variability, stressful life events, and the emergence of depressive symptomatology during the menopausal transition. Menopause.

[57] Mugavero MJ, Raper JL, Reif S, Whetten K, Leserman J, Thielman NM, Pence BW (2009). Overload: impact of incident stressful events on antiretroviral medication adherence and virologic failure in a longitudinal, multisite human immunodeficiency virus cohort study. Psychosom Med.

[58] Woods NF, Mitchell ES, Percival DB, Smith-DiJulio K (2009). Is the menopausal transition stressful? Observations of perceived stress from the Seattle midlife Women’s health study. Menopause.

[59] Freeman WE, Sammel MD, Boorman DW, Zhang R (2014). Longitudinal pattern of depressive symptoms around natural menopause. JAMA Psychiatry.

[60] Allsworth JE, Zierler S, Krieger N, Harlow BL (2001). Ovarian function in late reproductive years in relation to lifetime experiences of abuse. Epidemiology.

[61] Allsworth JE, Zierler S, Lapane KKL, Krieger N, Hogan JW, Harlow BL (2004). Longitudinal study of the inception of perimenopasue in relation to lifetime history of sexual or physical violence. J Epidemiol Community Health.

[62] Sternfeld B, Guthrie KA, Ensrud KE (2014). Efficacy of exercise for menopausal symptoms: a randomized controlled trial. Menopause.

[63] Lanza di Scalea T, Matthews KA, Avis NE, Thurston RC, Brown C, Harlow S, Bromberger JT (2012). Role stress, role reward, and mental health in a multiethnic sample of midlife women: results from the study of Women's health across the nation (SWAN). J Women's Health (Larchmt).

